# Utility of Fine Needle Aspiration Cytology in the Evaluation of Lymphadenopathy

**DOI:** 10.7759/cureus.11990

**Published:** 2020-12-09

**Authors:** Atif A Hashmi, Samreen Naz, Omer Ahmed, Syed Rafay Yaqeen, Muhammad Irfan, Anwar Kamal, Naveen Faridi

**Affiliations:** 1 Pathology, Liaquat National Hospital and Medical College, Karachi, PAK; 2 Internal Medicine, Liaquat National Hospital and Medical College, Karachi, PAK; 3 Internal Medicine, Baqai Medical University, Karachi, PAK; 4 Statistics, Liaquat National Hospital and Medical College, Karachi, PAK

**Keywords:** fine needle aspiration cytology (fnac), lymphadenopathy, tuberculosis (tb), reactive lymphadenitis, tuberculous (tb) lymphadenitis, metastatic carcinoma, lymphoproliferative disorder, hodgkin’s lymphoma, cytology

## Abstract

Introduction

Fine needle aspiration cytology (FNAC) is a quick, effective and relatively inexpensive technique to evaluate the visibly accessible superficial masses. As cervical, axillary and inguinal lymphadenopathies are commonly encountered clinical problems, in this study, we evaluated the utility of FNAC for assessment of lymphadenopathy.

Methods

A retrospective observational study was conducted in the Department of Cytopathology, Liaquat National Hospital and Medical College, over the duration of three years. A total of 559 cases were included in the study that underwent FNAC. After palpation, two to three passes were performed with a 22-23 gauge needle along with a plunger for FNAC. The obtained material was spread on three slides that were then stained with hematoxylin and eosin (H & E), Papanicolaou (PAP), and Diff-Quik methods. The remaining material was used for cell block preparation.

Results

The mean age of the patients was 37.05±18.03 years. In 98.7% of cases, the material was adequate for a satisfactory cytological examination. The most common site of FNAC was the cervical lymph node and tuberculous lymphadenitis (37%) was the most common diagnosis on FNAC, followed by reactive lymphadenitis (27.2%). Reactive lymphadenitis was seen more frequently in the younger age group (<15 years), whereas metastatic carcinoma was more commonly seen in the older age group (>50 years). Tuberculous lymphadenitis was noted more frequently in the middle age group (16-35 years). Moreover, tuberculous lymphadenitis was noted more commonly in cervical lymph nodes, while metastatic carcinoma was more frequently observed in axillary and inguinal lymph node FNACs.

Conclusion

FNAC is a quick and reliable method to categorize the cause of lymphadenopathy into reactive, inflammatory/infectious, metastatic, and lymphoproliferative, avoiding the necessity of an incisional/trucut biopsy. Moreover, age, gender, and site of involvement are useful predictors of the cause of lymphadenopathy. We noted that in the younger age group, reactive lymphadenitis was more common, whereas tuberculous lymphadenitis and metastatic carcinoma were more frequent in middle and older age groups, respectively. On a similar note, tuberculous lymphadenitis was more frequent in cervical lymph nodes than axillary and inguinal lymph nodes, while metastatic carcinoma was more common in these latter two sites.

## Introduction

Lymphadenopathy of cervical, axillary, and inguinal regions is a common clinical presentation in day-to-day clinical practice. There are diverse etiologies of enlarged lymph nodes, ranging from benign reactive lymphadenopathy (viral or bacterial infections), including tuberculosis (TB) to metastatic carcinomas and lymphomas. Fine needle aspiration cytology (FNAC) without radiological guidance is a quick, effective and relatively inexpensive technique to evaluate visibly accessible superficial masses [1-3]. Although the diagnostic material obtained by FNAC is considered of inferior quality than trucut/core needle biopsy, especially for the diagnosis of lymphomas, however, there are many advantages of FNAC over trucut biopsy. First, it’s an office procedure and there is no specific need for any prior hematological workup. Second, material adequacy can be checked quickly, and repeat FNAC can be done at the same time. Moreover, FNAC has an extremely low complication rate.

The utility of diagnostic cytopathology is rapidly increasing in laboratory diagnostics [4,5]. Apart from rapid turnover time, the obtained material can be used for molecular studies. As cervical, axillary and inguinal lymphadenopathies are commonly encountered clinical problems, in this study, we evaluated the utility of FNAC for assessment of lymphadenopathy.

## Materials and methods

A retrospective observational study was conducted in the Department of Cytopathology, Liaquat National Hospital and Medical College (LNH & MC). The duration of the study was three years from January 2016 until December 2018. A total of 559 cases were included in the study that underwent FNAC. The FNAC procedure was performed in the laboratory procedure room of the Cytopathology department, LNH. The FNAC procedure was done without radiological guidance for palpable lymph nodes of cervical, axillary, and inguinal regions. After palpation, two to three passes were performed with a 22-23 gauge needle along with a plunger for FNAC. The obtained material was spread on three slides that were then stained with hematoxylin and eosin (H & E), Papanicolaou (PAP), and Diff-Quik methods. The remaining material was used for cell block preparation. After the FNAC procedure was performed, two slides (H & E and Diff-Quik) were stained immediately and they were checked for material adequacy. If the diagnostic material was not present on the slides, another attempt of FNAC was performed. If the material was still inadequate, the fourth attempt of FNAC was performed after a gap of one day. The maximum number of FNAC attempts were four. The FNAC slides along with cell block slides were first screened by cytotechnologists and then finally examined and reported by cytopathologists. 

Data analysis was performed using Statistical Package for Social Sciences (Version 26.0, IBM Inc., Armonk, USA). Fisher's exact test was used to check the association. P-values ≤ 0.05 were considered as significant.

## Results

The mean age of the patients was 37.05±18.03 years and the majority of the patients were females (64%). In 98.7% of cases, the material was adequate for a satisfactory cytological examination. The most common site of FNAC was a cervical lymph node and tuberculous lymphadenitis (37%), followed by reactive lymphadenitis (27.2%) was the most frequent diagnosis on FNAC. Descriptive statistics of the population under study are shown in Table 1.

**Table 1:** Clinicopathological features of population under study SD: standard deviation; LN: lymph node; FNAC: fine needle aspiration cytology

Clinicopathological feature	Frequency (%)
Gender	
Male	201(36)
Female	358(64)
Age (years)	
Mean±SD	37.05±18.03
Age groups	
≤15 years	44(7.9)
16–35 years	257(46)
36–50 years	126(22.5)
>50 years	132(23.6)
Site of LN enlargement	
Cervical	469(83.9)
Axillary	78(14)
Inguinal	12(2.1)
Diagnosis on FNAC	
Tuberculous lymphadenitis	207(37)
Abscess	40(7.2)
Reactive lymphadenitis	152(27.2)
Lymphoproliferative disorder	40(7.2)
Metastatic carcinoma	106(19)
Necrotizing lymphadenitis	7(1.3)
Inadequate	7(1.3)

Metastatic carcinoma and lymphoproliferative disorder were significantly more commonly seen in males, whereas reactive lymphadenitis was more commonly seen in females (Table 2).

**Table 2:** Association of gender with the diagnosis on fine needle aspiration cytology Fisher's exact test was applied. *p-Value significant as <0.05. FNAC: fine needle aspiration cytology

Diagnosis on FNAC	Frequency (%)		p-Value
	Gender		
	Male	Female	
Tuberculous lymphadenitis	73(36.7)	134(38)	<0.0001*
Abscess	8(4)	32(9.1)	
Reactive lymphadenitis	44(22.1)	108(30.6)	
Lymphoproliferative disorder	24(12.1)	16(4.5)	
Metastatic carcinoma	49(24.6)	57(16.1)	
Necrotizing lymphadenitis	1(0.5)	6(1.7)	

Reactive lymphadenitis was seen more frequently in the younger age group (<15 years), whereas metastatic carcinoma was more commonly seen in the older age group (>50 years). Tuberculous lymphadenitis was noted more frequently in the middle age group (16-35 years) as shown in Table 3.

**Table 3:** Association of age groups with diagnosis on fine needle aspiration cytology Fisher's exact test was applied. *p-Value significant as <0.05. FNAC: fine needle aspiration cytology

Diagnosis on FNAC	Frequency (%)				p-Value
	Age groups				
	≤15 years	16–35 years	36–50 years	>50 years	
Tuberculous lymphadenitis	15(34.9)	135(52.9)	34(27.4)	23(17.7)	<0.0001*
Abscess	0(0)	23(9)	9(7.3)	8(6.2)	
Reactive lymphadenitis	25(58.1)	67(26.3)	36(29)	24(18.5)	
Lymphoproliferative disorder	0(0)	15(5.9)	9(7.3)	16(12.3)	
Metastatic carcinoma	2(4.7)	10(3.9)	36(29)	58(44.6)	
Necrotizing lymphadenitis	1(2.3)	5(2)	0(0)	1(0.8)	

Table 4 shows the association of site of nodal involvement with the diagnosis on FNAC. Tuberculous lymphadenitis was noted more commonly in cervical lymph nodes, while metastatic carcinoma was more frequently observed in axillary and inguinal lymph node FNACs.

**Table 4:** Association of site of nodal involvement with diagnosis on fine needle aspiration cytology Fisher's exact test was applied. *p-Value significant as <0.05. FNAC: fine needle aspiration cytology

Diagnosis on FNAC	Frequency (%)			p-Value
	Site of lymph node enlargement			
	Cervical	Axillary	Inguinal	
Tuberculous lymphadenitis	186(40.2)	18(23.4)	3(25)	<0.0001*
Abscess	30(6.5)	9(11.7)	1(8.3)	
Reactive lymphadenitis	136(29.4)	16(20.8)	0(0)	
Lymphoproliferative disorder	38(8.2)	0(0)	2(16.7)	
Metastatic carcinoma	68(14.7)	32(41.6)	6(50)	
Necrotizing lymphadenitis	5(1.1)	2(2.6)	0(0)	

Out of 40 cases diagnosed as a lymphoproliferative disorder on FNAC, a trucut/excision biopsy was performed on 19 cases. Of these 19 cases, four cases were diagnosed with Hodgkin's lymphoma, whereas 15 cases were diagnosed with non-Hodgkin's lymphoma.

## Discussion

In this study, we found that FNAC is a highly diagnostic technique in the evaluation of superficial lymphadenopathy of cervical, axillary, and inguinal regions. We also noted that tuberculous lymphadenitis was the most common reason for lymphadenopathy. Furthermore, in the younger age group, reactive lymphadenopathy was more common, in contrast to the older age group where metastatic carcinoma was more frequent.

In our study, we observed that the categorization of the cause of lymphadenopathy into reactive, inflammatory, metastatic, and lymphoproliferative could be reliably done by FNAC. A high sensitivity (71.4%) and specificity (91.5%) of FNAC was reported in previous studies for the evaluation of lymphadenopathy [6]. For patients with a known histologically proven malignancy in whom a subsequent enlargement of lymph node occurs, a cytological diagnosis of metastasis helps in avoiding unwanted surgical biopsy for confirming metastasis. In patients without a previous diagnosis of malignancy, FNAC not only confirms metastasis but in most circumstances gives a clue regarding the site of the primary. Furthermore, if cell block material is sufficient, immunohistochemical (IHC) studies can reliably identify the primary site of origin. In cases of lymphoproliferative diagnosis on FNAC, the cytological diagnosis should be followed by histological evaluation for accurate classification of lymphoma and grading. In our study, 19 cases were diagnosed with a lymphoproliferative disorder, all of them were finally diagnosed with either Hodgkin's or non-Hodgkin's lymphoma on histology.

**Figure 1:**
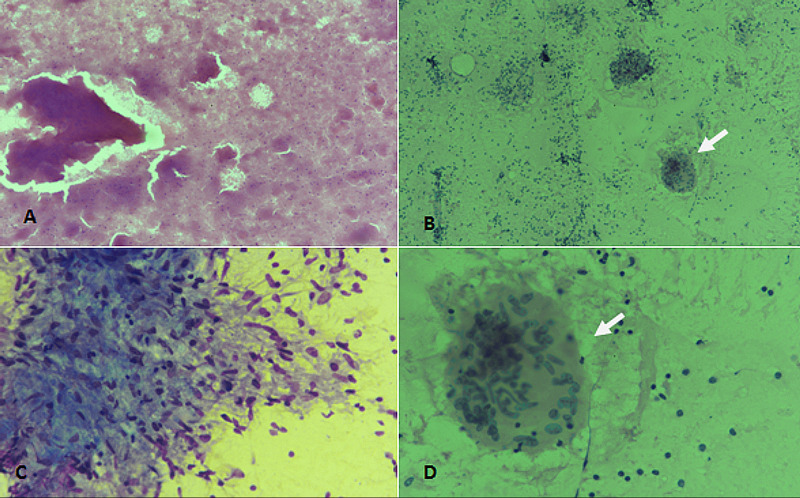
Tuberculous lymphadenitis. (A) H & E staining at 100× magnification showing caseous necrosis. (B) PAP staining at 100× magnification depicting Langhans type giant cell (arrow) with degenerated leucocytes in the background. (C) Diff-Quik staining at 400× magnification revealing epithelioid granulomas. (D) PAP staining at 400× magnification showing Langhans giant cell (arrow). H & E: hematoxylin and eosin; PAP: Papanicolaou

Tuberculous lymphadenitis was the most common cytological diagnosis on FNAC in our study. Cytologically, tuberculous lymphadenitis is characterized by caseation necrosis, epithelioid granulomas, and Langhans type giant cells (Figure 1).

A study evaluated 550 consecutive cases of TB diagnosed on FNAC. They noted that caseous necrosis with degenerated inflammatory cells in the background was the most common cytological pattern. They also found that cervical lymph nodes were the most common site and maximum incidence was in the third decade [7]. We also noted a higher frequency of TB in cervical lymph nodes with the most common occurrence in the 16-35 years age group. Another cytomorphological study investigating lymphadenopathy reported TB to be the most common cause of lymphadenopathy (44.02%), followed by reactive lymphadenitis (42.64%) and metastatic lesions (9.4%) [8].

**Figure 2:**
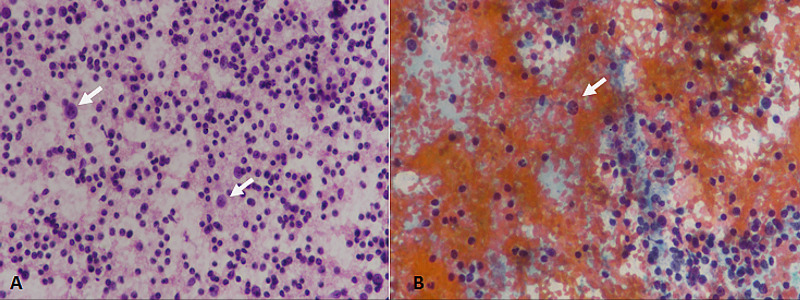
Reactive lymphadenitis. (A) H & E staining at 400× magnification depicting lymphoid cells with varying degrees of maturation. Most of the cells are small lymphocytes with some large-sized immunoblasts (arrows). (B) PAP staining at 400× magnification depicting variable size lymphoid cells with some immunoblasts (arrow). H & E: hematoxylin and eosin; PAP: Papanicolaou

Cytological analysis of reactive lymph node reveals lymphoid cells with variable maturation intermixed with immunoblasts, and tingible body macrophages (Figure 2).

Reactive lymphadenopathy was the second most common cause of lymphadenopathy in our study and was more frequently noted in the younger age group. These findings are consistent with other studies [8].

**Figure 3:**
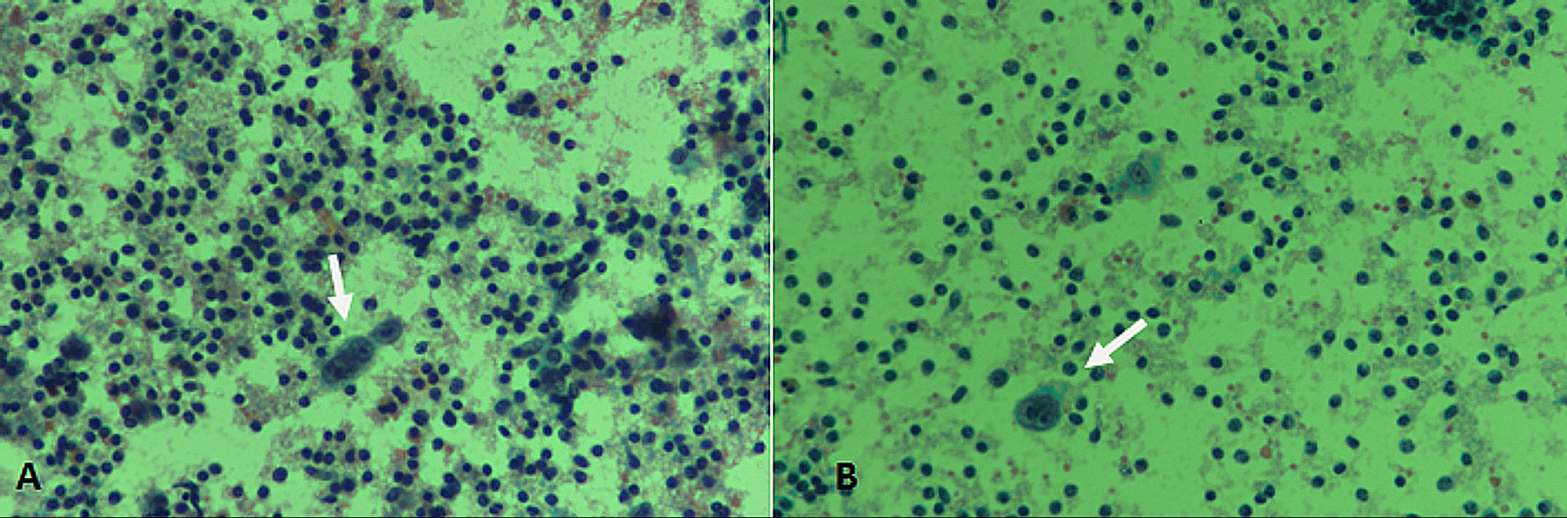
Hodgkin’s lymphoma. (A) & (B) PAP staining at 400× magnification showing large atypical Reed–Sternberg cells (arrows), with a reactive lymphoid background. PAP: Papanicolaou

Although, definite characterization of lymphomas is not possible on FNAC, however, diagnosis of Hodgkin's lymphoma can be done. Hodgkin’s lymphoma is characterized by characteristic Reed-Sternberg cells with a reactive cellular background (Figure 3).

**Figure 4:**
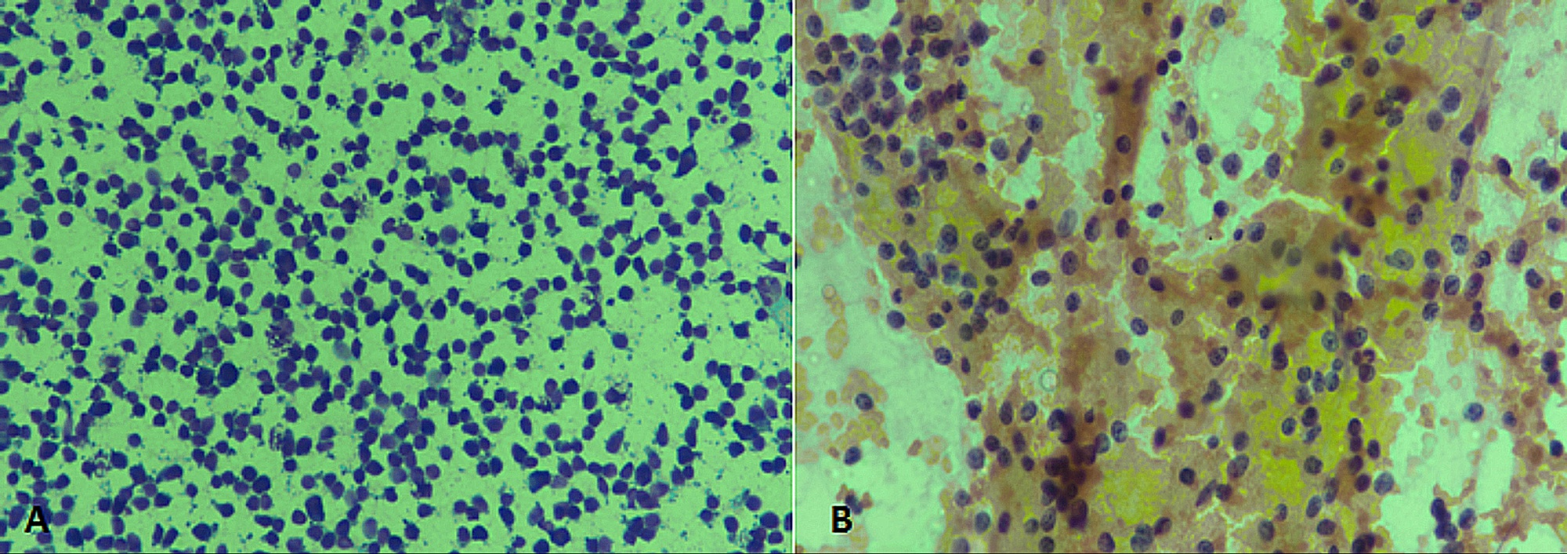
Non-Hodgkin’s lymphoma. (A) Diff-Quik staining at 400× magnification revealing a monotonous population of large atypical cells. (B) PAP staining at 400× magnification depicting scattered large atypical cells with scant cytoplasm. PAP: Papanicolaou

Alternatively, a monotonous population of lymphoid cells suggests non-Hodgkin's lymphoma (Figure 4).

**Figure 5:**
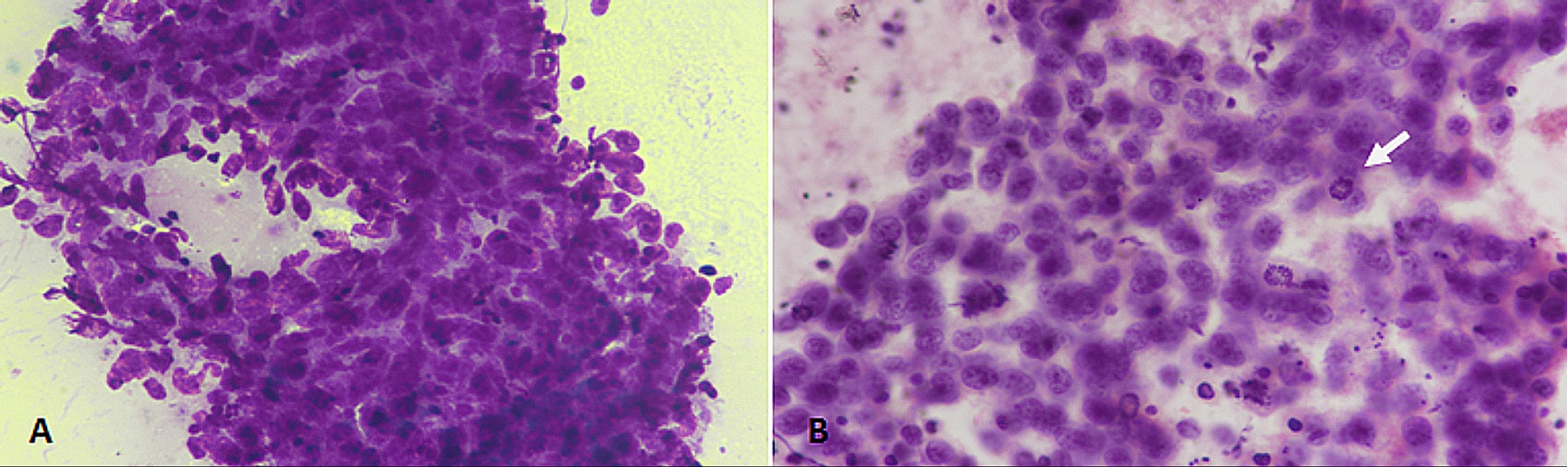
Metastatic carcinoma. (A) Diff-Quik staining at 400× magnification showing clusters of large atypical cells with hyperchromatic nuclei. (B) H & E staining at 400× magnification revealing loose cohesive clusters of large atypical cells. Mitotic figures are also evident (arrow). H & E: hematoxylin and eosin

FNAC is an important tool for metastatic workup, especially in cases of breast carcinoma and head and neck squamous cell carcinoma. A study evaluating metastatic lymphadenopathy by FNAC reported that the supraclavicular lymph node was the most common site of metastatic lymphadenopathy and squamous cell carcinoma was the most common type of metastatic carcinoma [9]. In our study, 19% of lymphadenopathy cases had a cytomorphological diagnosis of metastatic carcinoma, and a significant association of this diagnosis was noted with older age. Metastatic carcinoma on cytology is characterized by cohesive clusters of atypical cells (Figure 5).

There were a few limitations to our study. First, the biopsy was not performed in all these cases to determine the sensitivity, specificity, and diagnostic accuracy of FNAC. Second, clinical and radiological information of the patients was not available. In addition, the retrospective study design introduces biases secondary to confounding factors that cannot be controlled. 

## Conclusions

FNAC is an important tool for evaluating lymphadenopathy. Categorization of the cause of lymphadenopathy into reactive, inflammatory/infectious, metastatic, and lymphoproliferative disorder can be reliably done by FNAC, avoiding the need for truct/excisional biopsy. Furthermore, age, gender, and site of lymphadenopathy also provide useful information in predicting the cause of lymphadenopathy, as reactive lymphadenitis was more common in the younger age group, tuberculous lymphadenitis in middle age, and metastatic carcinoma in the older age group. Similarly, tuberculous lymphadenitis was more commonly noted in cervical lymph nodes, whereas metastatic carcinoma was more frequent in axillary and inguinal lymph nodes.
